# A histone H4 lysine 20 methyltransferase couples environmental cues to sensory neuron control of developmental plasticity

**DOI:** 10.1242/dev.145722

**Published:** 2017-04-01

**Authors:** Colin E. Delaney, Albert T. Chen, Jacqueline V. Graniel, Kathleen J. Dumas, Patrick J. Hu

**Affiliations:** 1 Departments of Cell and Developmental Biology, University of Michigan Medical School, Ann Arbor, MI 48109, USA; 2 Internal Medicine, University of Michigan Medical School, Ann Arbor, MI 48109, USA; 3 Institute of Gerontology, University of Michigan Medical School, Ann Arbor, MI 48109, USA

**Keywords:** *C. elegans*, Dauer, Dosage compensation, H4K20, Insulin-like peptides, FoxO

## Abstract

Animals change developmental fates in response to external cues. In the nematode *Caenorhabditis elegans*, unfavorable environmental conditions induce a state of diapause known as dauer by inhibiting the conserved DAF-2 insulin-like signaling (ILS) pathway through incompletely understood mechanisms. We have previously established a role for the *C. elegans* dosage compensation protein DPY-21 in the control of dauer arrest and DAF-2 ILS. Here, we show that the histone H4 lysine 20 methyltransferase SET-4, which also influences dosage compensation, promotes dauer arrest in part by repressing the X-linked *ins-9* gene, which encodes a new agonist insulin-like peptide (ILP) expressed specifically in the paired ASI sensory neurons that are required for dauer bypass. *ins-9* repression in dauer-constitutive mutants requires DPY-21, SET-4 and the FoxO transcription factor DAF-16, which is the main target of DAF-2 ILS. By contrast, autosomal genes encoding major agonist ILPs that promote reproductive development are not repressed by DPY-21, SET-4 or DAF-16/FoxO. Our results implicate SET-4 as a sensory rheostat that reinforces developmental fates in response to environmental cues by modulating autocrine and paracrine DAF-2 ILS.

## Abstract (precis)

**Summary:** The *C. elegans* histone methyltransferase SET-4 acts to link environmental signals to diapause through regulation of the X-linked insulin-like peptide gene *ins-9* in sensory neurons.

## INTRODUCTION

To maintain evolutionary fitness, organisms must react appropriately to environmental cues. The free-living nematode *Caenorhabditis elegans* has evolved a developmental strategy to optimize survival in changing environments. Under replete conditions, larvae progress through four stages (L1-L4) to become reproductive adults. In adverse conditions such as overcrowding, heat or food scarcity, larvae arrest in an alternative stage known as dauer. Adapted for survival in harsh environments, dauers are morphologically, metabolically and behaviorally distinct from reproductive L3 larvae. Improvement of ambient conditions induces dauer exit and resumption of reproductive development (Fielenbach and Antebi, 2008; Riddle, 1988). The dominant environmental cue that influences dauer arrest is a constitutively elaborated pheromone that indicates population density (Butcher et al., 2008; Golden and Riddle, 1982).

*C. elegans* dauer arrest has served as a useful paradigm for understanding the molecular basis of developmental plasticity. Genetic analysis has defined four conserved signaling pathways that promote reproductive development in favorable environments. The DAF-11 transmembrane guanylyl cyclase acts in chemosensory neurons to regulate dauer arrest through the cyclic nucleotide-gated channel subunits TAX-2 and TAX-4. Downstream of DAF-11, DAF-2 insulin receptor (InsR)- and DAF-7 transforming growth factor-β (TGFβ)-like pathways act in parallel to promote reproductive development by inhibiting the activities of the FoxO transcription factor DAF-16 and the SMAD transcription factor DAF-3, respectively. Distal to DAF-16/FoxO and DAF-3/SMAD, bile-acid-like steroid hormones known as dafachronic acids (DAs) promote reproductive development by regulating the activity of the conserved nuclear receptor DAF-12 (Fielenbach and Antebi, 2008).

Although genetic analysis has identified how components of the DAF-11, DAF-2/InsR, DAF-7/TGFβ and DAF-12 pathways interact to promote reproductive development in favorable conditions (Gottlieb and Ruvkun, 1994; Riddle et al., 1981; Thomas et al., 1993; Vowels and Thomas, 1992), the molecular nature of the upstream events that couple external cues to the activities of these pathways remains poorly understood. Laser ablation experiments demonstrated that the amphid sensory neurons are required for induction of dauer arrest by pheromone (Schackwitz et al., 1996; Vowels and Thomas, 1994). Indeed, the dauer-inhibitory ASI sensory neurons (Bargmann and Horvitz, 1991) are specific sites of expression of three insulin-like peptides (ILPs) that promote reproductive development through DAF-2/InsR (INS-4, INS-6 and DAF-28) (Chen and Baugh, 2014; Cornils et al., 2011; Hung et al., 2014; Li et al., 2003), as well as the DAF-7 TGFβ-like ligand that promotes reproductive development (Ren et al., 1996; Schackwitz et al., 1996). Furthermore, crude dauer pheromone reduces the expression of DAF-28 and DAF-7 in ASI (Li et al., 2003; Schackwitz et al., 1996), suggesting that pheromone induces dauer arrest at least in part by reducing the expression of agonist ligands in sensory neurons that regulate DAF-2/InsR and TGFβ-like signaling. How pheromone represses these ligands remains a mystery.

We have previously reported an unforeseen role for the *C. elegans* dosage compensation protein DPY-21 in promoting dauer arrest through inhibition of the DAF-2/InsR pathway (Dumas et al., 2013). DPY-21 is a component of the condensin-like dosage compensation complex (DCC) that equalizes X-linked gene expression between males and hermaphrodites by binding to both hermaphrodite X chromosomes during embryogenesis and repressing gene expression approximately twofold (Meyer, 2010; Yonker and Meyer, 2003). Here, we show that the conserved histone H4 lysine 20 (H4K20) methyltransferase SET-4, which also influences dosage compensation (Kramer et al., 2015; Vielle et al., 2012; Wells et al., 2012), promotes dauer arrest in a sex-specific manner by synergizing with DAF-16/FoxO to repress *ins-9*, an X-linked gene that encodes an ILP expressed specifically in ASI neurons (Chen and Baugh, 2014; Pierce et al., 2001). These findings reveal a sexually dimorphic role for regulators of histone H4K20 methylation in broadening the dynamic range of sensory responses to environmental cues that control developmental plasticity.

## RESULTS

### SET-4 acts through DAF-2 ILS to promote dauer arrest in a sex-specific manner

**Fig. 1.**
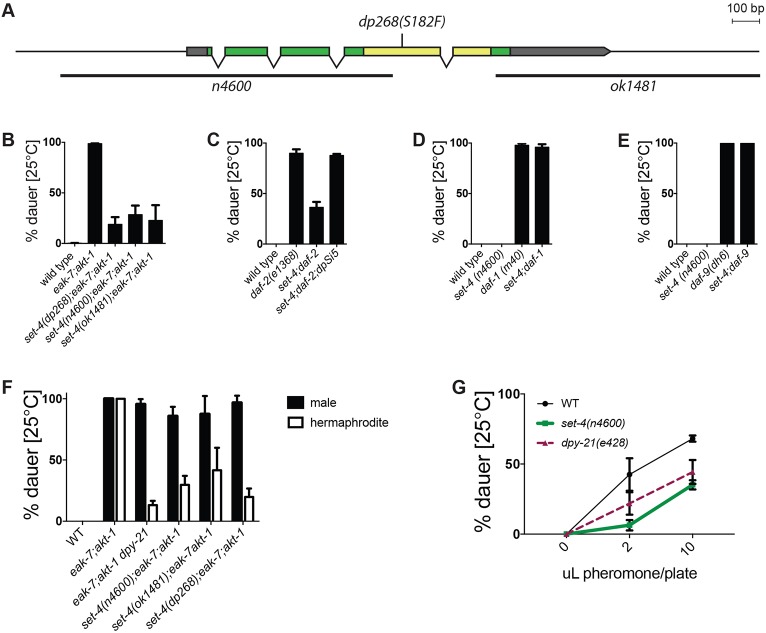
**SET-4 promotes dauer arrest.** (A) Schematic of the *set-4* locus and three mutant alleles. Exons are indicated as boxes, separated by introns. Gray, green and yellow denote untranslated regions, coding sequence and SET domain coding sequence, respectively. Deletions are indicated by black bars. (B) *set-4* mutations suppress the dauer-constitutive phenotype of *eak-7;akt-1* mutants [*n* (left to right)=236, 627, 389, 521, 638]. (C) *set-4(n4600)* suppresses the dauer-constitutive phenotype of *daf-2(e1368)* mutants and is rescued by the single-copy *set-4* transgene *dpSi5* (*n*=913, 1668, 1568, 1785). (D,E) *set-4* is dispensable for dauer arrest in (D) *daf-1(m40)* (*n*=2215, 2189, 1816, 1899) and (E) *daf-9(dh6)* mutants (*n*=1753, 1561, 2188, 2486). (F) *set-4* and *dpy-21* mutations suppress dauer arrest in XX hermaphrodites but not in XO males (*n*=567, 1330, 579, 1003, 1012, 700). (G) *set-4* and *dpy-21* mutations attenuate the response of wild-type animals to dauer pheromone [*n* (0 μl, 2 μl, 10 μl): wild type=542, 461, 544; *set-4*=413, 246, 387; *dpy-21*=334, 220, 275]. *set-4* versus wild type: *P*<0.01 by two-way ANOVA.

DAF-2/InsR promotes reproductive development by activating a conserved phosphoinositide 3-kinase (PI3K)/Akt pathway to inhibit DAF-16/FoxO (Murphy and Hu, 2013). The conserved protein EAK-7 acts in parallel to AKT-1 to inhibit nuclear DAF-16/FoxO activity (Alam et al., 2010). In contrast to *eak-7* and *akt-1* single mutants, which develop reproductively, *eak-7;akt-1* double mutant animals arrest as dauers in a DAF-16/FoxO-dependent manner (Alam et al., 2010). To identify new DAF-16/FoxO regulators, we performed a forward genetic screen for suppressors of the *eak-7;akt-1* dauer-constitutive phenotype (*seak*). The first *seak* mutants characterized harbored loss-of-function mutations in the dosage compensation gene *dpy-21* (Dumas et al., 2013)*. dpy-21* encodes a conserved component of the condensin-like dosage compensation complex (DCC) that binds to X chromosomes and represses X-linked gene expression (Meyer, 2010; Yonker and Meyer, 2003). One *seak* mutant strain contained a point mutation in *set-4*, which encodes a histone H4K20 methyltransferase homolog that influences dosage compensation (Vielle et al., 2012; Wells et al., 2012). *set-4(dp268)* is predicted to change the conserved SET domain catalytic residue serine 182 (Southall et al., 2014) to phenylalanine (Fig. 1A, Fig. S1A). In light of our findings on *dpy-21* (Dumas et al., 2013), we tested the possibility that *set-4(dp268)* was the causative *seak* mutation in this strain. After outcrossing removed all but two closely linked single nucleotide variants, *set-4(dp268)* suppressed dauer arrest to a similar extent to two independently derived *set-4* deletions, *n4600* (Andersen and Horvitz, 2007) and *ok1481* (Fig. 1B). Furthermore, an integrated single-copy *HA::set-4* transgene rescued dauer arrest in *set-4*(*n4600*) animals (Fig. 1C and Fig. S1B). Therefore, SET-4 promotes dauer arrest.

DPY-21 enhances dauer arrest by activating DAF-16/FoxO, indicating that it acts in the DAF-2/InsR pathway to regulate dauer formation (Dumas et al., 2013). To determine whether SET-4 functions in the DAF-2/InsR pathway, we tested the effect of *set-4* mutation on dauer-constitutive phenotypes caused by mutations in the *daf-2/InsR*, *daf-7/TGFβ* and *daf-12* pathways. *set-4* mutation suppressed the dauer-constitutive phenotypes of *daf-2(e1368)* mutants (Fig. 1C) as well as *akt-1(ok525)* and *eak-7(tm3188)* single mutants, which develop reproductively at 25°C but arrest as dauers at 27°C (Fig. S1C,D) (Ailion and Thomas, 2003; Alam et al., 2010; Hu et al., 2006). By contrast, *set-4* mutation had no effect on the dauer-constitutive phenotypes caused by mutations in *daf-1*, which encodes a type 1 TGFβ receptor homolog (Georgi et al., 1990), or *daf-8*, which encodes a SMAD homolog (Park et al., 2010) (Fig. 1D and Fig. S1E). Similarly, *set-4* mutation did not suppress dauer arrest in animals harboring mutations in *daf-9* or *daf-36*, which encode DA biosynthesis pathway components (Fig. 1E and Fig. S1F) (Gerisch et al., 2001; Jia et al., 2002; Rottiers et al., 2006). Therefore, SET-4 acts specifically in the DAF-2/InsR pathway to promote dauer arrest.

As previous reports link H4K20 methylation status to dosage compensation (Vielle et al., 2012; Wells et al., 2012) and DPY-21 promotes dauer arrest through dosage compensation (Dumas et al., 2013), we hypothesized that SET-4 may also regulate dauer arrest through dosage compensation. To test this, we determined the effect of *set-4* mutation on the dauer-constitutive phenotype of *eak-7;akt-1* double mutant hermaphrodites and males. If SET-4 promotes dauer arrest through the same mechanism as dosage compensation, then *set-4* mutation should suppress dauer in hermaphrodites but not in males, as the DCC is inactive in males (Meyer, 2010). *dpy-21* and *set-4* mutations suppressed the dauer-constitutive phenotype of *eak-7;akt-1* hermaphrodites but did not affect dauer arrest in males (Fig. 1F). Therefore, SET-4 may act through dosage compensation to control dauer arrest. We verified the role of SET-4 in dosage compensation by showing that *set-4* mutation suppressed lethality in *xol-1 sex-1* mutant males, which die due to inappropriate activation of dosage compensation (Dawes et al., 1999) (Fig. S1G).

In order to determine whether SET-4 plays a role in regulating dauer entry in wild-type animals in response to physiologic stimuli, we tested the ability of dauer pheromone to induce dauer arrest in wild-type and *set-4* mutant animals. Mutation of either *set-4* or *dpy-21* decreased the sensitivity of wild-type animals to pheromone (Fig. 1G). Therefore, SET-4 and DPY-21 promote dauer arrest in wild-type animals in response to increases in population density.

### SET-4 is a H4K20 methyltransferase

**Fig. 2.**
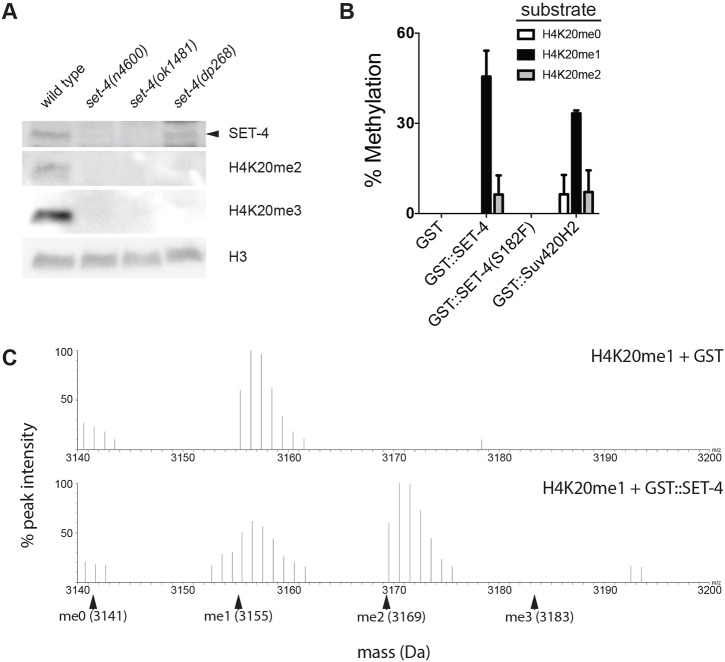
**SET-4 is a histone H4K20 methyltransferase.** (A) SET-4 promotes H4K20 methylation *in vivo*. Anti-SET-4, H4K20me2, H4K20me3 and H3 immunoblots of lysates from wild-type and *set-4* mutant animals are shown. SET-4 protein is indicated by the arrowhead. Images are representative of four biological replicates. (B) Recombinant GST-SET-4 fusion protein methylates H4K20me1 *in vitro*. Percentage methylation of H4K20 peptide substrates by GST proteins fused to wild-type SET-4, mutant SET-4(S182F) or human SET-4 ortholog SUV420H2 is shown. Data represent mean values from three biological replicates. (C) MALDI spectra illustrating conversion of H4K20me1 to H4K20me2 by GST-SET-4. Monoisotopic masses (protonated) of peptide substrates are indicated with arrowheads. Spectra are representative of three independent experiments.

The mammalian SET-4 ortholog SUV420H2 is a H4K20 methyltransferase (Schotta et al., 2004), and *C. elegans* SET-4 promotes H4K20 trimethylation (Vielle et al., 2012; Webster et al., 2013; Wells et al., 2012). We confirmed the requirement of SET-4 for H4K20 di- and trimethylation *in vivo* (Fig. 2A). Immunoblots showed no detectable SET-4 protein in *set-4(n4600)* and *set-4(ok1481)* backgrounds, consistent with these being strong loss-of-function alleles. SET-4 protein levels in *set-4(dp268)* are comparable with wild type (Fig. 2A). H4K20me2 and H4K20me3 levels are undetectable in all three *set-4* mutant backgrounds (Fig. 2A), suggesting that the S182F substitution in the SET domain abrogates catalytic activity. To test this possibility directly, we purified recombinant wild-type and mutant GST-SET-4 fusion proteins and tested their ability to methylate modified H4 peptides (H4K20me0, H4K20me1 and H4K20me2) *in vitro*. Mass spectrometry analysis revealed that both wild-type GST-SET-4 and GST-SUV420H2 were capable of converting H4K20me1 to H4K20me2 (Fig. 2B,C). Consistent with *in vitro* experiments using human SET-4 orthologs SUV420H1 and SUV420H2 (Southall et al., 2014; Wu et al., 2013), methylation was not detected with unmethylated or dimethylated substrates, nor were trimethylated products detected in any assay (Fig. 2B,C). It is possible that an enzyme distinct from SET-4 catalyzes H4K20 trimethylation *in vivo*. Alternatively, conversion of H4K20me2 to H4K20me3 by SET-4 *in vivo* may require a co-factor that is absent from our *in vitro* reactions.

GST-SET-4(S182F) did not methylate H4K20me1, indicating that the S182F mutation abolishes catalytic activity (Fig. S2). Given that *set-4(dp268)* suppresses dauer to a similar extent to the two deletion alleles (Fig. 1B), these data are consistent with SET-4 influencing dauer arrest through its conserved role in methylating H4K20.

### SET-4 acts in neurons to promote dauer arrest

**Fig. 3.**
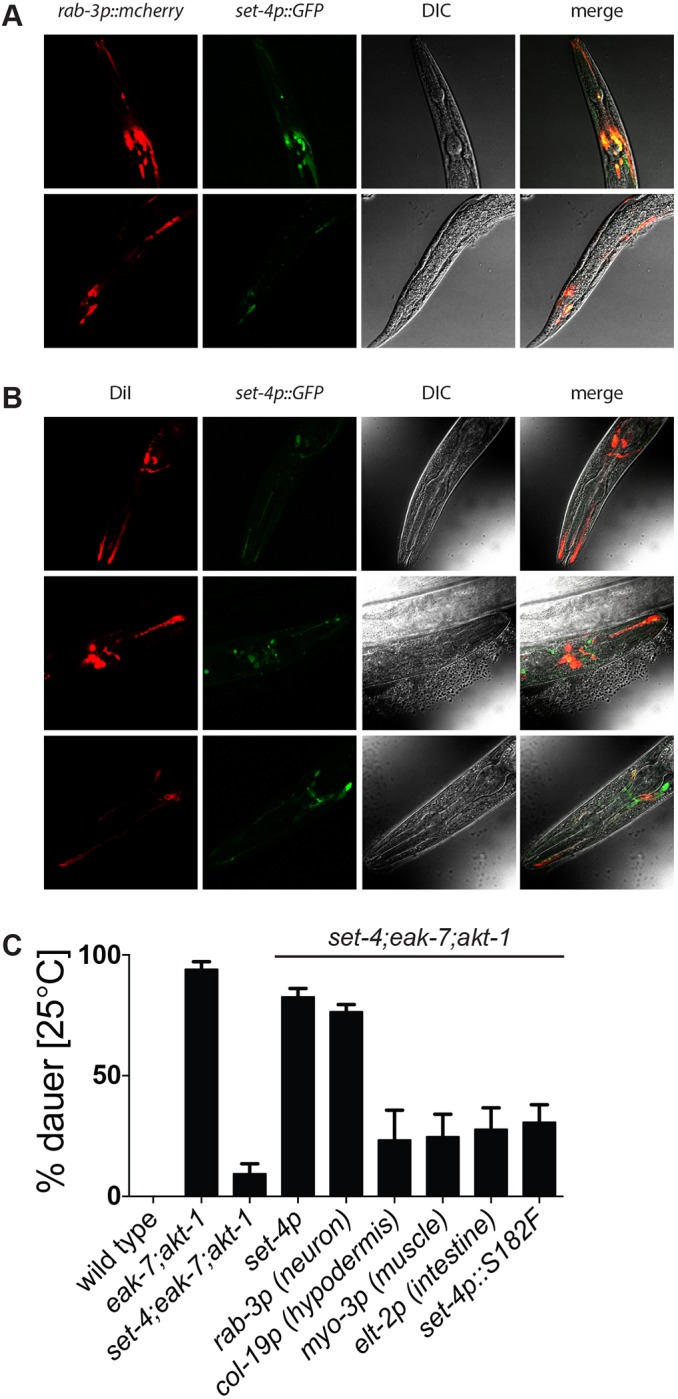
***set-4* acts in the nervous system to promote dauer arrest.** (A) Representative photomicrographs showing colocalization of GFP and mCherry in animals co-expressing *set-4p::gfp* and the pan-neuronal reporter *rab-3p::mcherry* (*n*=15). (B) Representative photomicrographs showing colocalization of red and green fluorescence in amphid neurons of *set-4p::gfp* transgenic animals exposed to DiI (*n*=21). (C) Rescue of dauer arrest in *set-4;eak-7;akt-1* mutants by *set-4* transgenes driven by native *set-4* or tissue-specific promoters (*n*=2328, 2143, 2058, 662, 811, 705, 936, 516, 541).

To determine where SET-4 is expressed, we generated strains expressing reporter genes under the control of *set-4* regulatory elements. Because two independent C-terminal SET-4::GFP translational fusions failed to rescue dauer arrest in *set-4* mutants, we generated strains expressing a *set-4p::*GFP promoter fusion to determine the spatiotemporal activity of the *set-4* promoter. *set-4p::*GFP transgenic animals expressed GFP broadly in embryos (Fig. S3). Post-embryonically, we detected fluorescence predominantly in the head and tail regions of the animal, in a pattern consistent with neuronal expression. To confirm this, we established a transgenic strain that co-expressed *set-4p::*GFP and mCherry driven by the pan-neuronal *rab-3* promoter (Nonet et al., 1997). At all developmental stages interrogated, we observed colocalization of green and red fluorescence (Fig. 3A), consistent with somatic *set-4p::*GFP expression being predominantly neuronal. We did not observe significant GFP expression in intestine, body wall muscle or hypodermis.

As the amphid sensory neurons play a crucial role in regulating dauer arrest (Bargmann and Horvitz, 1991; Schackwitz et al., 1996; Vowels and Thomas, 1994), we interrogated them for expression of *set-4p::*GFP. Amphid neurons possess ciliary dendrites that are in direct contact with the environment and can be labeled with the lipophilic dye DiI (Starich et al., 1995). The extent of colocalization of green and red fluorescence in *set-4p::*GFP transgenic animals exposed to DiI (Fig. 3B) reveals that the *set-4* promoter is active in amphid sensory neurons as well as in other cells.

To test whether neuronal expression of *set-4* is functionally important for dauer arrest, we generated tissue-specific *set-4* transgenes and tested them for the ability to rescue dauer arrest in *set-4* mutants. The neuronal *rab-3p::set-4* transgene rescued dauer formation to a similar extent to a *set-4* transgene driven by its native promoter (Fig. 3C). By contrast, intestine-, hypodermis- and muscle-specific *set-4* transgenes did not rescue dauer arrest to a greater extent than a transgene expressing the *set-4(dp268)* mutant*.* Taken together, these data indicate that SET-4 functions in the nervous system to promote dauer arrest.

### Transcriptome-wide influences of DPY-21 and SET-4 on dauer regulation

We previously showed that the DCC component DPY-21 promotes DAF-16/FoxO activity (Dumas et al., 2013). To gain broader insight into how DPY-21 and SET-4 control dauer arrest, we performed whole-transcriptome profiling to compare genome-wide regulatory influences (henceforth referred to as the ‘regulome’) of DPY-21 and SET-4 to those of the key transcription factors controlling dauer arrest in *eak-7;akt-1* animals, DAF-16/FoxO and the nuclear receptor DAF-12 (Alam et al., 2010). We identified genes differentially expressed between wild-type and *eak-7;akt-1* double mutant animals [fold change ≥1.5 and false discovery rate (FDR) <0.05]. We then compared the transcriptomes of *eak-7;akt-1* double mutants with those of *eak-7;akt-1* animals harboring mutations in *dpy-21*, *set-4*, *daf-16/FoxO* or *daf-12*, and identified genes that are differentially expressed in the opposite direction as in wild-type relative to *eak-7;akt-1* (Table S1). Regulomes were validated by comparison with published data where possible (see below).

We defined the SET-4 dauer regulome by identifying 333 genes common to *set-4(n4600)* and *set-4(dp268)* regulomes (Table S1). Comparison of this gene set with SET-4-regulated genes identified in wild-type embryos and L3 larvae (Kramer et al., 2015) revealed minimal overlap [one gene (MTCE.34) among 94 regulated by SET-4 in embryos, and one gene (B0511.11) among 18 SET-4-regulated genes in L3 larvae]. This lack of concordance could be due to differences in genetic background (*eak-7;akt-1* versus wild-type), developmental stage (early L2 larvae versus embryos/L3 larvae) and/or ambient temperature (25°C versus 20°C).

**Fig. 4.**
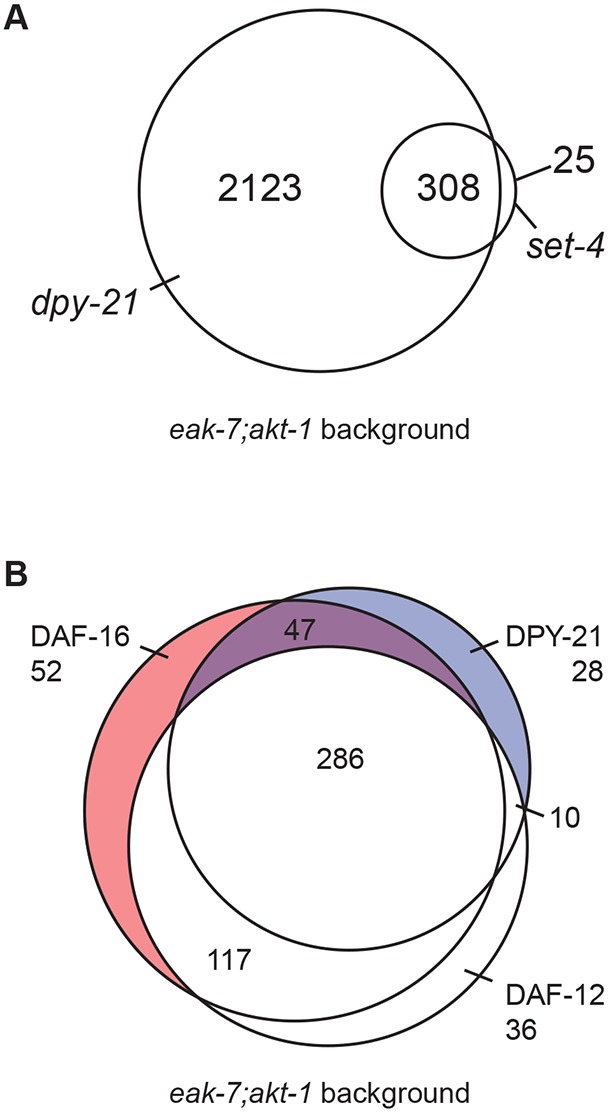
**Whole-transcriptome profiling defines genes coordinately regulated by DPY-21/SET-4 and DAF-16/FoxO.** (A) Venn diagram of genes regulated by SET-4 and DPY-21. Three-hundred and eight genes are coordinately regulated by SET-4 and DPY-21. (B) Venn diagram of X-linked genes regulated by DPY-21, DAF-16/FoxO and DAF-12. Forty-seven genes coordinately regulated by DPY-21 and DAF-16/FoxO but not by DAF-12 are depicted in purple and listed in Table S5. Data represent the aggregate of five biological replicate samples, each from thousands of progeny with no fewer than 200 animals per sample.

A similar analysis with *eak-7;akt-1 dpy-21* mutants revealed 2431 genes that comprise the DPY-21 dauer regulome (Table S1). To validate the DPY-21 regulome, we found significant overlap between the set of 700 X-linked genes differentially expressed in *eak-7;akt-1 dpy-21* versus *eak-7;akt-1* animals with the 374 X-linked genes subject to dosage compensation in embryos (Jans et al., 2009) (119 genes; Fig. S4A and Table S2; *P*=2.1e^−26^). Three hundred and eight of the 333 genes that make up the SET-4 dauer regulome (92.5%) are also part of the DPY-21 dauer regulome (Fig. 4A and Table S1), suggesting that a functional relationship between DPY-21 and SET-4 may exist in post-embryonic dauer regulation.

The DAF-16/FoxO regulome consists of 2957 genes (Table S1). This gene set overlapped significantly with both the 469-gene young adult DAF-16/FoxO regulome (Chen et al., 2015) (203 common genes; Fig. S4B and Table S3, *P*=3.2e^−115^) as well as the 1116-gene dauer regulome defined using *daf-7* TGFβ-like pathway mutants (Liu et al., 2004) (522 common genes; Fig. S4C and Table S4, *P*<1e^−100^). Furthermore, 65 of the 132 genes regulated by the DAF-7 TGFβ-like pathway that contained at least one upstream DAF-16/FoxO-binding motif (Liu et al., 2004) were part of the DAF-16/FoxO regulome [Fig. S4D and Table S4 (bold text), *P*=6.5e^−42^]. Most genes in the DAF-16/FoxO regulome (2282 of 2957 genes; 77.2%) are also regulated by DAF-12 (Fig. 4B and Table S1). Over two-thirds of genes that comprise both DAF-16/FoxO (2041 of 2957 genes; 69.0%; Table S1) and DAF-12 (1804 of 2556 genes; 70.6%; Table S1) regulomes are concordantly regulated by DPY-21.

### The X-linked *ins-9* gene is repressed by DPY-21, SET-4 and DAF-16/FoxO

Based on genetic epistasis experiments (Dumas et al., 2013) (Fig. 1), we hypothesized that DPY-21 and SET-4 influence DAF-16/FoxO activity through repression of X-linked genes. Moreover, in light of the neuronal site of action of SET-4 (Fig. 3C) and its expression in amphid sensory neurons (Fig. 3B), we speculated that key dauer regulatory genes subject to dosage compensation might function in a signaling capacity in the nervous system, upstream of DAF-12. Therefore, we examined the set of X-linked genes common to DPY-21 and DAF-16/FoxO regulomes that were not regulated by DAF-12, which acts downstream in the dauer regulatory cascade (Fielenbach and Antebi, 2008; Schaedel et al., 2012). This filtering strategy defined a set of 47 X-linked genes coordinately regulated by DPY-21 and DAF-16/FoxO but not influenced by *daf-12* mutation (Fig. 4B; Table S5).

**Fig. 5.**
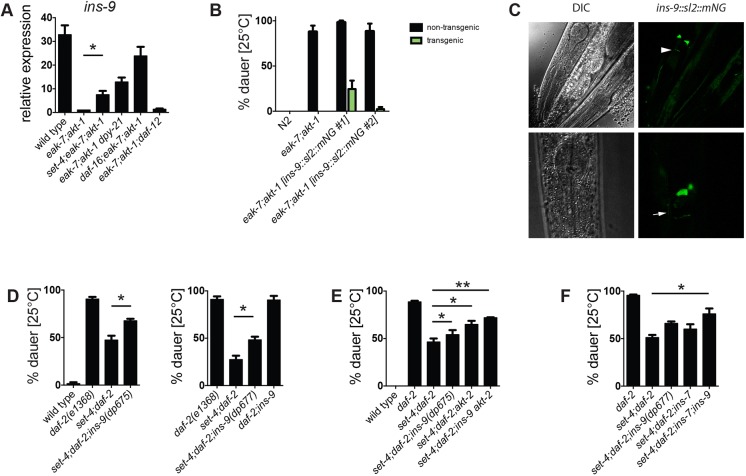
**SET-4 promotes dauer arrest by repressing the X-linked *ins-9* gene.** (A) *ins-9* is repressed by DPY-21, SET-4 and DAF-16/FoxO but not DAF-12. Results are the mean±s.e.m. of five experiments. (B) *ins-9* overexpression suppresses dauer arrest in *eak-7;akt-1* animals. Non-transgenic (NT) and transgenic (T) progeny of transgenic hermaphrodites are shown. *n*=1200, 1522, 718/156 (NT/T), 702/210 (NT/T). (C) An *ins-9::SL2::mNG* transgene is expressed specifically in ASI sensory neurons. The arrowhead indicates the top of the nerve ring; the arrow indicates the ASI axon. Representative images are shown (*n*=20). (D) Two *ins-9* nonsense mutations partially rescue dauer arrest in *set-4;daf-2* mutants. *n*=957, 1205, 1398 (left); *n*=892, 1028, 897 (right). (E) *ins-9* and *akt-2* mutations rescue dauer arrest in an additive fashion in *set-4;daf-2* mutants (*n*=949, 1281, 1334, 1524, 1333, 1478). (F) *ins-9* and *ins-7* mutations rescue dauer arrest in an additive fashion in *set-4;daf-2* mutants (*n*=1396, 1251, 754, 827, 1327). *P<0.05, **P<0.01.

Among these X-linked genes was *ins-9*, which encodes one of 40 *C. elegans* ILPs (Pierce et al., 2001). Transcriptome profiling revealed that *ins-9* is silenced in *eak-7;akt-1* animals in a manner that requires both *daf-16/FoxO* and *dpy-21* (Table S2). We verified this by qPCR; *ins-9* expression was reduced more than 30-fold in *eak-7;akt-1* double mutants compared with wild-type animals (Fig. 5A). Full repression required DAF-16/FoxO as well as DPY-21 and SET-4, but was independent of *daf-12*. Notably, a *daf-16/FoxO*-null mutation did not restore *ins-9* transcript levels to wild-type levels. Furthermore, mutation of either *dpy-21* or *set-4* increased *ins-9* expression by substantially greater than twofold (Fig. 5A; 7.5-fold increase in *set-4;eak-7;akt-1* versus *eak-7;akt-1*; 13.5-fold increase in *eak-7;akt-1 dpy-21* versus *eak-7;akt-1*). Taken together, these observations suggest that DAF-16/FoxO and DPY-21/SET-4 act synergistically to repress *ins-9*.

### *ins-9* overexpression phenocopies dauer suppression caused by *dpy-21* or *set-4* mutation

INS-9 was an attractive candidate regulator of dauer arrest and DAF-2 ILS, as multiple ILPs have been shown to control dauer arrest through DAF-2/InsR (Cornils et al., 2011; Fernandes de Abreu et al., 2014; Hung et al., 2014; Li et al., 2003; Murphy et al., 2003; Pierce et al., 2001). If derepression of *ins-9* contributes to suppression of *eak-7;akt-1* dauer arrest in dosage compensation mutants, then *ins-9* overexpression should phenocopy dauer suppression caused by *set-4* or *dpy-21* mutation. We tested this by establishing transgenic strains harboring a polycistronic construct that permitted expression of both *ins-9* and *mNeonGreen* (Shaner et al., 2013) fused to an SL2 leader sequence (Blumenthal, 2005) under the control of native *ins-9* 5′ and 3′ regulatory elements (*ins-9::SL2::mNG*). In two independent transgenic lines, *ins-9* overexpression (indicated by mNeonGreen detection) suppressed the dauer-constitutive phenotype of *eak-7;akt-1* double mutants (Fig. 5B). Therefore, *ins-9* overexpression recapitulated the phenotype caused by *set-4* and *dpy-21* mutations (Dumas et al., 2013) (Fig. 1B). These data are consistent with INS-9 acting as an agonist DAF-2/InsR ligand.

### *ins-9* is expressed specifically in a single pair of amphid neurons

Previous studies using reporters driven by the *ins-9* promoter suggested that *ins-9* is expressed in the ASI and ASJ amphid neurons, as well as in additional tissues (Chen and Baugh, 2014; Pierce et al., 2001; Ritter et al., 2013). By contrast, in transgenic L2 larvae expressing *ins-9::SL2::mNG*, we consistently observed green fluorescence solely in one pair of sensory neurons. In animals in which neuronal morphology was discernable, we identified the fluorescent cells as the ASI amphid neurons (Fig. 5C). We did not observe fluorescence in more than one pair of amphid neurons in any animal, nor did we detect fluorescence in other neurons or tissues. As *ins-9::SL2::mNG* contains genomic elements from the *ins-9* locus that are missing from other reporters in the literature (Chen and Baugh, 2014; Pierce et al., 2001; Ritter et al., 2013), these observed patterns of expression are likely to be physiologically relevant.

### *ins-9* and *akt-2* are required for suppression of dauer arrest by *set-4* mutation

To determine the extent to which *ins-9* derepression contributes to dauer suppression in *set-4* mutants, we tested the ability of *set-4* to suppress the dauer-constitutive phenotype of *daf-2/InsR* mutants in wild-type and *ins-9* loss-of-function backgrounds. We generated strong loss-of-function *ins-9* alleles using CRISPR/Cas9 genome editing (Paix et al., 2015). Two probable null alleles, *dp675* and *dp677*, have nonsense mutations in the F-peptide region of *ins-9* that lies N-terminal to the functional B and A peptides (Pierce et al., 2001) (Fig. S5). Although neither allele induced dauer arrest in a wild-type background, both *ins-9(dp675)* and *ins-9(dp677)* partially rescued dauer arrest in *set-4;daf-2* double mutants (Fig. 5D), indicating that dauer suppression caused by *set-4* mutation is due in part to de-repression of *ins-9*.

We previously showed that the X-linked gene *akt-2* is required for dauer suppression caused by *dpy-21* mutation (Dumas et al., 2013). We verified that *akt-2* transcripts increase two-fold in *eak-7;akt-1* double mutants when *set-4* or *dpy-21* is mutated (Fig. S6A). Similar to *ins-9* mutation, *akt-2* mutation also partially rescued dauer arrest in animals lacking *set-4* (Fig. 5E), and the phenotypic effects of *ins-9* and *akt-2* mutations on dauer arrest may be additive (Fig. 5E). However, *set-4;daf-2;ins-9 akt-2* compound mutant animals still did not undergo dauer arrest to the same extent as *daf-2* single mutant animals (Fig. 5E), indicating that regulation of additional genes may contribute to dauer arrest.

### The autosomal *ins-7* gene contributes to suppression of dauer arrest by *set-4* mutation

As the only X-linked *ins* gene, *ins-9* is the sole *ins* gene subject to direct regulation by dosage compensation or other X-chromosome-specific mechanisms of gene regulation. However, it is conceivable that other *ins* genes could contribute to dauer regulation through indirect effects on their expression. Genes encoding three agonist ILPs, INS-4, INS-6 and DAF-28, are expressed in the ASI sensory neurons and have established roles in inhibiting dauer arrest and promoting reproductive development (Cornils et al., 2011; Hung et al., 2014; Li et al., 2003). To determine whether regulation of *ins-4*, *ins-6* and/or *daf-28* contributes to dauer suppression in this context, we measured *ins-4*, *ins-6* and *daf-28* transcript levels in wild-type, *eak-7;akt-1* double mutant and *eak-7;akt-1* triple mutants with reduced DPY-21 or SET-4 activity. None of these genes was repressed in *eak-7;akt-1* double mutants compared with wild-type animals, nor did loss of *set-4* or *dpy-21* cause significant increases in their expression (Fig. S6B-D). Therefore, neither DPY-21 nor SET-4 influences dauer arrest through regulation of *ins-4*, *ins-6* and *daf-28* expression.

To determine whether other ILPs could contribute to dauer regulation by DPY-21 or SET-4, we searched the set of genes common to DAF-16/FoxO and DPY-21 regulomes for *ins* genes. Seven *ins* genes are concordantly regulated by DAF-16/FoxO and DPY-21 based on FPKM counts from whole-transcriptome data (Table S6). Two genes encoding putative antagonist ILPs, *ins-20* and *ins-11* (Fernandes de Abreu et al., 2014), are repressed by DAF-16/FoxO and DPY-21; however, an increase in their expression in *dpy-21* mutants would be expected to enhance, rather than suppress, dauer arrest. Similarly, two *ins* genes encoding agonist ILPs, *ins-33* and *ins-35* (Fernandes de Abreu et al., 2014; Michaelson et al., 2010), are induced by DAF-16/FoxO and DPY-21; a decrease in their expression in *dpy-21* mutants would also be expected to enhance dauer arrest if changes in their expression were functionally important in dauer regulation. DAF-16/FoxO and DPY-21 induce the expression of two *ins* genes of unknown function, *ins-16* and *ins-29* (Fernandes de Abreu et al., 2014)*.* Finally, *ins-7*, which encodes an agonist ILP (Murphy et al., 2007, 2003), is repressed by DAF-16/FoxO and DPY-21 (Murphy et al., 2007, 2003) (Table S6). As *ins-7* and *ins-9* both encode agonist ILPs, are concordantly regulated by DAF-16/FoxO and DPY-21, and have been shown to influence dauer arrest (Murphy et al., 2007, 2003) (Fig. 5C-E), we tested *ins-7* for a role in promoting reproductive development in *dpy-21* and *set-4* mutants. We verified *ins-7* repression by DAF-16/FoxO, SET-4 and DPY-21 using qPCR (Fig. S6E). The *ins-7(tm1907)* deletion allele partially rescued dauer in *set-4;daf-2* animals and may have an additive effect with *ins-9* mutation on dauer suppression (Fig. 5F). Thus, SET-4 may influence dauer arrest through both the direct repression of X-linked genes such as *ins-9* and *akt-2* and perhaps the indirect regulation of autosomal dauer regulatory genes such as *ins-7*.

## DISCUSSION

**Fig. 6.**
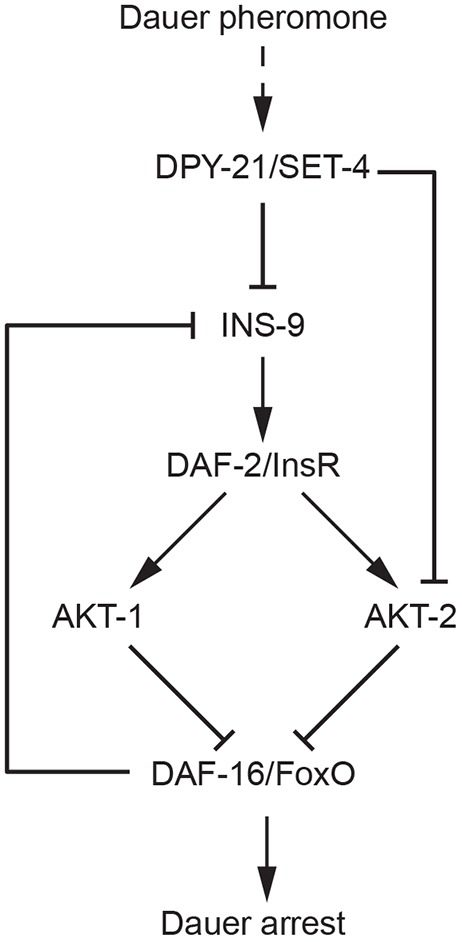
**Hypothetical model of dauer regulation by pheromone through DPY-21/SET-4 and DAF-2 ILS.** DPY-21 and SET-4 act in concert to promote transduction of pheromone cues by repressing X-linked genes encoding the DAF-2/InsR agonist INS-9 and the serine/threonine kinase AKT-2, resulting in increased DAF-16 activation and subsequent dauer arrest.

Although much is known about the conserved signaling pathways that control *C. elegans* dauer arrest, how these pathways are regulated by upstream inputs is poorly understood. In the present study, we have established a framework for understanding how DPY-21 and SET-4 promote dauer arrest in the context of reduced DAF-2 ILS. Specifically, we have discovered that the conserved H4K20 methyltransferase SET-4 acts in the nervous system to promote dauer arrest. It does so, in part, by synergizing with DAF-16/FoxO to repress the X-linked insulin-like peptide gene *ins-9*. We hypothesize that SET-4 and DPY-21 act similarly to repress *ins-9* and *akt-2* directly, thus attenuating DAF-2 ILS and promoting dauer arrest through activation of DAF-16/FoxO (Fig. 6).

Although INS-9 has been predicted to function as an agonist ILP based both on structural models that indicate similarity to the agonist ILPs INS-4, INS-6 and DAF-28 (Pierce et al., 2001) and on expression changes upon starvation and feeding of larvae (Chen and Baugh, 2014), analysis of existing *ins-9* mutants has not revealed phenotypes consistent with this (Fernandes de Abreu et al., 2014). This may be due to *ins-9(tm3618)* not being a strong loss-of-function allele. By contrast, our analysis of transgenic animals overexpressing *ins-9* (Fig. 5B) and mutant animals harboring nonsense *ins-9* alleles (Fig. 5D-F) provides the first experimental evidence demonstrating that INS-9 is an agonist ILP.

Several ILPs have been implicated in dauer regulation (Cornils et al., 2011; Fernandes de Abreu et al., 2014; Hung et al., 2014; Li et al., 2003; Murphy et al., 2003; Pierce et al., 2001). However, the mechanistic basis for how environmental cues regulate ILPs remains obscure. The initial events that control dauer arrest through DAF-2 ILS likely take place in the amphid sensory neurons, which are required for dauer formation in response to pheromone (Schackwitz et al., 1996; Vowels and Thomas, 1994). Indeed, genes encoding INS-4, INS-6 and DAF-28, which collectively play a major role in promoting reproductive development through DAF-2 ILS (Cornils et al., 2011; Hung et al., 2014; Li et al., 2003), are expressed in ASI (Chen and Baugh, 2014; Cornils et al., 2011; Hung et al., 2014; Li et al., 2003), and the transcription of *ins-6* and *daf-28* is inhibited by dauer pheromone through unknown mechanisms (Cornils et al., 2011; Li et al., 2003). Our finding that DPY-21 and SET-4 influence dauer arrest in part by repressing the X-linked *ins-9* gene provides a potential mechanistic link between a dauer-regulatory environmental cue and an ILP expressed in sensory neurons that control the dauer decision.

An intriguing but incompletely understood aspect of dauer morphogenesis is the mechanistic basis for the commitment of larvae to either the reproductive or dauer developmental fate. Assays in which larvae are shifted between favorable and unfavorable conditions at different times after hatching indicate the existence of commitment points beyond which animals develop reproductively or arrest as dauers regardless of ambient conditions (Golden and Riddle, 1984; Schaedel et al., 2012). Commitment to reproductive development correlates temporally with the activation of a feed-forward loop amplifying organismal DA biosynthesis through DA-dependent induction of DAF-9 expression in the hypodermis (Schaedel et al., 2012). However, the XXX cells (Ohkura et al., 2003), which are thought to be the sole source of DA biosynthesis prior to the commitment point (Schaedel et al., 2012), are not in direct contact with the environment. Therefore, they must receive upstream inputs from sensory neurons that convey information about ambient conditions.

Our finding that DPY-21 and SET-4 synergize with DAF-16/FoxO to repress *ins-9* is consistent with a hypothetical model in which INS-9 may function as a key node in an autocrine feed-forward loop in the ASI sensory neurons that reinforces levels of its own expression in response to changing environments, upstream of DA biosynthesis in the XXX cells and hypodermis. In replete conditions, *ins-9* expression in ASI is expected to lead to activation of DAF-2 ILS and inhibition of DAF-16/FoxO. As DAF-16/FoxO inhibits *ins-9* expression, decreased DAF-16/FoxO activity would lead to increased *ins-9* expression, which would presumably lead to further activation of DAF-2 ILS and inhibition of DAF-16/FoxO, both in an autocrine fashion in ASI as well as in other cells that express DAF-2/InsR. In the context of increased population density, pheromone would promote *ins-9* repression through DPY-21 and SET-4, and reduce autocrine and paracrine engagement of DAF-2/InsR, resulting in DAF-16/FoxO activation, further repression of *ins-9* and dauer arrest (Fig. 6). The effect of DPY-21 and SET-4 would not be limited to sensory neurons, as they would also act in other cells responding to INS-9 to control their sensitivity to ILPs by repressing *akt-2* (Dumas et al., 2013) (Fig. 6 and Fig. S6A). In addition, *ins-9* regulation may also be amplified through other ILPs such as INS-7, which functions in a feed-forward loop in adults to coordinate DAF-16/FoxO activity throughout the animal (Murphy et al., 2007).

## MATERIALS AND METHODS

### *C. elegans* strains and maintenance

Mutant alleles are listed in the supplementary Materials and Methods. Compound mutants were generated using standard protocols. All animals were maintained on nematode growth media (NGM) plates seeded with *E. coli* OP50 using standard techniques. Strains are available upon request.

### Dauer arrest assays

Dauer arrest assays were performed as previously described (Hu et al., 2006). *daf-9(dh6)* mutant animals were propagated on NGM plates supplemented with 10 nM Δ^7^-DA, then transferred to NGM plates for egglays as previously described (Dumas et al., 2013). For male dauer assays, males were crossed to isogenic L4 hermaphrodites, and the gender of dauer progeny was determined after dauer exit.

### Dauer pheromone assays

Dauer pheromone was prepared as previously described (Golden and Riddle, 1982; Schroeder and Flatt, 2014). Details are provided in the supplementary Materials and Methods.

### Generation of transgenic strains

Details pertaining to the generation of reporter constructs and transgenic strains are provided in the supplementary Materials and Methods.

### CRISPR/Cas9-based mutagenesis

*ins-9(dp675)* and *ins-9(dp677)* were generated using recombinant crRNA and tracrRNA (Dharmacon) and Cas9 (PNA Bio) as described previously (Paix et al., 2015). See Table S7 for sequences of guide RNAs and repair oligonucleotides.

### RNA isolation

Greater than 200 gravid hermaphrodites were allowed to lay eggs for 6 h at 20°C and then removed. Eggs were transferred to 25°C for 24 h. Larvae were harvested, washed once in M9 buffer and once in water, and resuspended in TRIzol (Invitrogen). After five sequential freeze-thaws, RNA was extracted using chloroform. Extracted RNA was purified using a Direct-zol RNA Miniprep Kit (Zymo Research).

### qPCR

cDNA was synthesized with oligo-dT priming using the SuperScript III First Strand Synthesis Kit (Invitrogen). The equivalent of 10 ng of starting RNA was used as template in a 15 μl reaction using the Quantitect SYBRgreen qPCR Kit (Qiagen). Reactions were performed in a RotorGene 6000 (Corbett Research) and results analyzed using RotorGene 6000 Software (version 1.7). Samples were normalized to *pmp-3* expression prior to comparison between groups (Hoogewijs et al., 2008). See Table S8 for primer sequences. Relative expression was calculated as described (Nolan et al., 2006).

### Confocal microscopy

Animals were mounted on slides layered with a thin 3% agarose pad containing 25 mM sodium azide. Images were captured on a Leica Inverted SP5X Confocal Microscope (Leica) using LAS AF software.

### RNA-seq analysis

Whole-transcriptome profiling was performed by the University of Michigan DNA Sequencing Core as previously described (Chen et al., 2015) using 100 ng input RNA per sample. Samples were barcoded and multiplexed, and 100-nucleotide paired-end sequencing was performed using an Illumina HiSeq 2000 sequencer and Version 4 reagents. Five experimental replicates were analyzed. Correlation coefficients between replicates and genotypes are shown in Table S9.

Annotated gene expression data output from CuffDiff v2.2.1 (Trapnell et al., 2013) was read into R version 3.2.1 (2015-06-18; The R Foundation for Statistical Computing; http://www.r-project.org/) for six comparisons: *eak-7;akt-1* compared with: (1) wild type, (2) *daf-16(mu86);eak-7;akt-1*, (3) *daf-12;eak-7;akt-1*, (4) set*-4(n4600);eak-7;akt-1*, (5) *set-4(dp268);eak-7;akt-1* and (6) *dpy-21;eak-7;akt-1*. We filtered genes using the following criteria: (1) status=‘OK’ for wild type versus *eak-7;akt-1*, (2) fold change (FC) ≥1.5 or FC ≤1/1.5 for wild type versus *eak-7;akt-1* and (3) FDR <0.05 for at least two separate comparisons.

DAF-16 targets were defined as those filtered genes that also met (1) status=‘OK’ for *eak-7;akt-1* versus *daf-16;eak-7;akt-1* and (2) FC ≥1.5 for *eak-7;akt-1* versus *daf-16;eak-7;akt-1* in the opposite direction to wild type versus *eak-7;akt-1*. DPY-21, SET-4 and DAF-12 targets were determined in an analogous fashion. For SET-4, we generated a list of SET-4 targets that showed FC ≥1.5 or FC ≤1/1.5 for both *set-4* alleles, and a list that showed these changes for either one or both *set-4* alleles. Lists of overlapping targets were then generated from these target lists.

The significance of overlap with dosage-compensated X-linked genes (Jans et al., 2009), strongly regulated dauer genes (Liu et al., 2004) and DAF-16 targets in the *daf-2(e1370)* background (Chen et al., 2015) was calculated using a hypergeometric distribution, assuming 5863 X-linked transcripts and 46233 genome-wide transcripts in *C. elegans* detected in our RNA-seq analysis (by either the UCSCce10 reference transcriptome or *de novo* transcript assembly). If necessary, common WormBase Gene identifiers were downloaded from WormBase version WS250 (intermine.wormbase.org).

### Immunoblotting and antibodies

To generate protein lysates, animals were washed in M9, then in sterile water. Pelleted animals were resuspended in equal volumes of worm lysis buffer (Webster et al., 2013), incubated at 85°C for 5 min, then sonicated on ice for two cycles of 30 s each at 70% power using a Sonic Dismembrator Model 100 (Fisher Scientific). Homogenates were quantified using a DC Protein Quantification Kit (BioRad). Protein (50 μg per lane) was loaded using Criterion systems (BioRad) and transferred to Immobilon Psq (Millipore). Details pertaining to antibodies are provided in the supplementary Materials and Methods. Membranes were blocked with 5% milk in TBS+0.5% Tween 20. Antibodies were diluted in Western Blocking Solution (Sigma) prior to incubation with membranes. Blots were washed with TBS+0.5% Tween 20. Signal was detected by ECL (Pierce).

### Histone methyltransferase assay

*set-4* cDNAs were amplified from RNA isolated from wild-type or *set-4(dp268)* mutant animals. Human *Suv420H2* cDNA was obtained from Origene. Clones were ligated into pGEX4T1 vector (GE Healthcare). Protein expression was induced overnight at 16°C with 0.1 mM IPTG in BL21-CodonPlus(DE3)-RIPL cells (Agilent Technologies) grown in Terrific Broth (Invitrogen)+4% glycerol (Sigma). Cells were disrupted using a Sonic Dismembrator Model 100 sonicator (Fisher Scientific), with four cycles of ten 1 s on/off pulses of 10-30% intensity. Lysates were cleared by centrifugation at 20,000 ***g*** for 5 min and incubated with Glutathione-Sepharose beads (GE Healthcare) rotating overnight at 4°C. Expression of recombinant protein was confirmed by Coomassie staining and anti-GST immunoblot. Beads bound to recombinant protein were incubated in 10 mM Tris (pH 8.0), 2 μM β-mercaptoethanol, and 7 mM S-adenosylmethionine (Sigma) in the presence of 2 mM substrate peptide corresponding to amino acids 8-30 of *C. elegans* histone H4 (AnaSpec) rotating for 4 h at 30°C. Reactions were analyzed using a Waters MicroMass MALDI-TOF mass spectrometer and analyzed with MassLynx software. Ratios of peak heights corresponding to reactant and product peptide were calculated to define percent conversion.

### Male rescue assay

Mated gravid hermaphrodites were placed on NGM plates to lay eggs for 24 h at 20°C. Egglayers were removed, and the numbers of hatchlings and eggs were counted. After 72 h, the animals were moved to 4°C to slow their movement. Male rescue was calculated as the ratio of live males to total eggs laid. Each experiment was performed in triplicate.

### Statistics

Two-tailed Student's *t*-test was used to measure significance in experiments unless otherwise noted. Data are presented as the average±standard error of the mean (s.e.m.) of at least three biological replicates, each replicate performed in triplicate. *n* values for dauer assays are listed from left to right.
